# Effects of Hot-Hydrostatic Canned Extrusion on the Stock Utilization, Microstructure and Mechanical Properties of TiBw/TC4 Composites with Quasi-Continuous Network

**DOI:** 10.3390/ma10111227

**Published:** 2017-10-25

**Authors:** Yangju Feng, Bing Li, Guorong Cui, Wencong Zhang

**Affiliations:** School of Materials Science and Engineering, Harbin Institute of Technology at Weihai, Weihai 264209, China; 13B909079@hit.edu.cn (Y.F.); libinghitmse@163.com (B.L.); zwinc@hitwh.edu.cn (W.Z.)

**Keywords:** TiBw/TC4 composites, canned extrusion, stock utilization, microstructure, mechanical properties

## Abstract

In-situ TiB whisker-reinforced Ti–6Al–4V (TC4) titanium matrix composites (TiBw/TC4) with quasi-continuous networks were successfully fabricated by vacuum hot-pressing sintering. The effects of the hot-hydrostatic canned extrusion on stock utilization, microstructure and mechanical properties of the TiBw/TC4 composites were investigated. It was satisfactory that the utilization of composites could be obviously improved by canned extrusion compared to that extruded without canned extrusion. The microstructure results showed that after canned extrusion the grain was refined and the TiB whiskers were distributed from a random array state to a state in which the whiskers were distributed along the extrusion direction. The properties testing results revealed that the tensile strength, the hardness and the ductility of the composites all significantly improved after extrusion due to the grain refinement and orientation of the TiB whisker caused by extrusion. Tensile fracture results showed that when the TiB whiskers were randomly distributed only part of them played a role in strengthening the matrix during the deformation process (as-sintered composites), while when the TiB whiskers were oriented all whiskers could strengthen the matrix during the tensile testing process (as-extruded composites).

## 1. Introduction

Titanium matrix composites (TMCs) not only possess the advantages of titanium alloys such as high specific strength, good weldability and excellent corrosion resistance [1,2,3,4,5] but can also address the shortcomings of titanium alloys such as not performing well in higher temperature and abrasion resistance [6]. Thus, TMCs are quite sought-after in aerospace, military and automotive [7,8,9,10]. In-situ synthesized TiB whiskers have been considered as almost the best reinforcement due to their excellent thermodynamic stability and similar coefficient of thermal expansion to the titanium matrix [6,11,12]. In-situ TiB whisker-reinforced TMCs fabricated by powder metallurgy (PM) methods have become more attractive due to the intrinsic advantages of PM methods such as low waste of raw material, composition homogenization and near-net forming [8,13,14]. For the purpose of solving the extreme brittleness for TMCs with homogeneous reinforcement distribution fabricated by the conventional PM processes [3,12,14,15] had proposed a new structure with network reinforcement distribution and achieved a gratifying result. It is well known that traditional follow-up processing such as extrusion, forging and rolling, could improve the microstructure and the mechanical properties of the TMCs and form a simple shape [15]. Most TMCs must be deformed in hot conditions due to the low deformation limit, large deformation resistance and being easy-to-crack at room temperature [16,17]. It is difficult for the hot working of TMCs because of the high deformation temperature and large flow stress. It is inevitable that the TiB whisker would be broken during the deformation process [18,19]. The cracks produced by the broken TiB whisker would reduce the performance of the TMCs. Taking this issue into account, hot hydrostatic canned extrusion is very suitable for the hot processing of TiB whisker-reinforced TMCs. On the one hand, three-dimensional (3D) compressive stress during the extrusion process could promptly remedy the cracks caused by the fractured TiB whiskers. On the other hand, the can could be used as a suitable lubricant during the extrusion process to solve the problem of the large deformation resistance of titanium alloy.

In addition to the performance of TMCs, their cost has attracted more and more attention. The high cost largely limits the application of the TMCs. The utilization of materials determines the cost to a great extent. In this paper, the stock utilization is discussed whether cans are used during the extrusion process or not.

In summary, the TiBw/TC4 composites with quasi-continuous networks were hot hydrostatic extruded with/without steel can. The stock utilization, microstructure and mechanical properties of as-extruded composites are discussed in this paper, which may be able to provide some guidance for the subsequent processing of the titanium alloys and TMCs. Moreover, it is significant for the promotion of mass production of titanium alloys/TMCs bars.

## 2. Experimental Procedures

**Figure 1:**
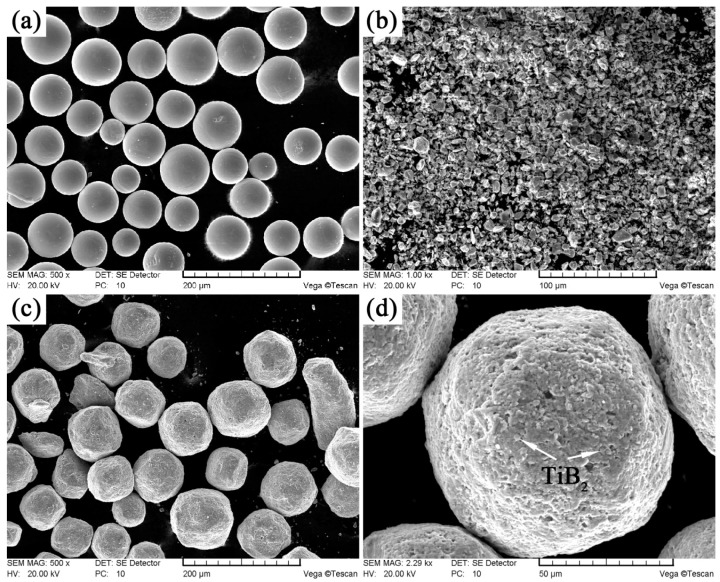
Scanning electron microscopy (SEM) micrographs of raw materials: (**a**) TC4 powders; (**b**) TiB_2_ powders, lower-energy milled powders with low magnification (**c**); and high magnification (**d**).

**Figure 2:**
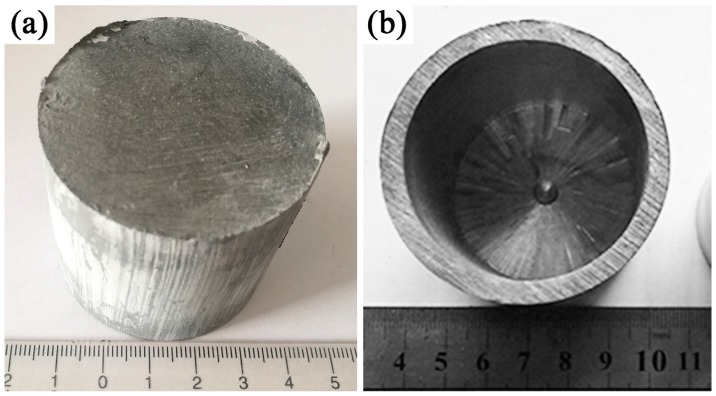
As-sintered TiBw/TC4 composites (**a**) and 45# steel can (**b**).

The spherical TC4 powders (Ti–6.4Al–4.1V, Figure 1a) and prismatic TiB_2_ powders (Figure 1b) were chosen as the raw materials for the production of the in-situ synthesized 2.5 vol % TiBw/TC4 composites. The mass fraction ratio of TC4 powder/TiB_2_ powder was 97 to 3 according to reference [14]. The average size (diameter) of spherical TC4 powders and TiB_2_ powders were approximately 115 µm and 4 µm, respectively. The both powders were low energy milled at a speed of 100 rpm for a period of 6 h under argon atmosphere. The mass ratio of steel ball to powders was 5:1. Subsequently, the mixed powder (Figure 1c,d) was sintered in a graphite mold in vacuum of 10^−2^ Pa at 1200 °C under a pressure of 25 MPa for 45 min. TiB phase was in-situ synthesized through the reaction of TiB_2_ and titanium surrounding TiB_2_. After the process of heat preservation and pressure maintaining, the composites were cooled at a very slow furnace-cooling rate. The dimensions of as-sintered composites were 50 mm in diameter and 45 mm in height (Figure 2a).

The as-sintered composites were weld-sealed into a 45# steel can (with the dimensions of 52 mm in outside diameter, 40 mm in inside diameter, 50 mm in height, as shown in Figure 2b), which was prepared in advance. Then the columnar billets were pre-sintered in a SX2-20-16 high temperature box furnace at 1150 °C for 0.5 h. After the sintering process, the billets were promptly extruded in a four-column hydraulic machine (HP3-315) (Tiankai, Nantong, China) with an extrusion ratio of 10.6:1 and the extrusion velocity of 10 mm/s followed by air cooling. The as-extruded bars were 16 mm in diameter and 500 mm in length. The oil based graphite was used as the lubricant during the extrusion process. Additionally, the composites were also prepared under identical condition for comparison without using can during the extrusion process.

Powder size was measured by laser particle size distribution instrument (BT-2003). Microstructure observation was conducted using scanning electron microscopy (SEM, Zeiss) (Zeiss, Jena, Germany). Before observation, metallographic sandpaper was first used to remove the mark of wire-electrode from the specimen. Then the samples were polished and etched in Kroll's solution (5 vol % HF + 10 vol % HNO_3_ + 85 vol % H_2_O) for 8~10 s. For tensile test, the specimens were cut along the extrusion direction using electric spark cutting and the gauge dimensions of the specimens were 4 mm×2 mm×15 mm. Before testing, the metallographic sandpaper was used to remove the mark of wire electrodes from the specimen. Then room temperature tensile tests were performed on an Instron-5569 universal testing machine (Instron, Boston, MA, America) at the constant crosshead speed of 0.5 mm/min (with a corresponding strain rate of 5.5 × 10^−4^ s^−1^). The extensometer was used during the tensile testing process for measuring the ductility. At least three samples were used for tensile test and the average value of strength and elongation was obtained by calculating arithmetic mean value. Vickers hardness was tested at the condition of 1 kg load for 10 s. In order to ensure the accuracy of the hardness data, at least five samples were tested and 20 points were randomly selected for each sample.

## 3. Results and Discussion

### 3.1. Phase Identification

**Figure 3:**
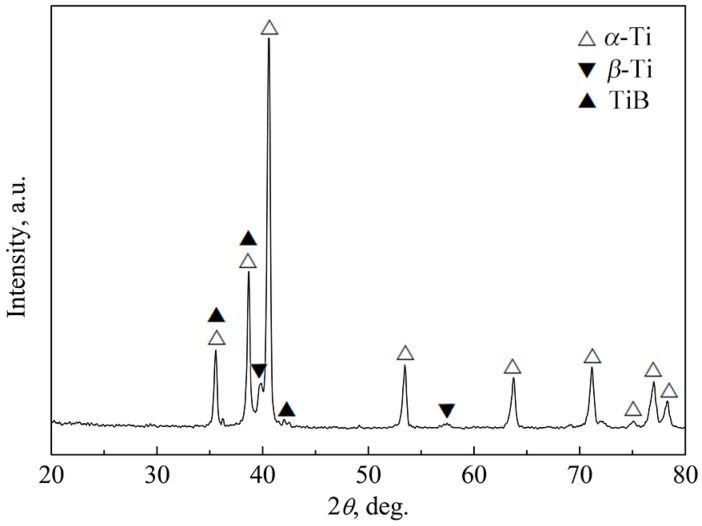
X-ray diffraction (XRD) patterns of the as-sintered 2.5 vol % TiBw/TC4 composites.

Figure 3 shows the X-ray diffraction (XRD) patterns of the as-sintered 2.5 vol % TiBw/TC4 composites. There were three kinds of phases in the composites, i.e., α-Ti, β-Ti and TiB. This result indicated that TiB phase could be in-situ synthesized through the reaction of TiB_2_ and Ti during the vacuum hot-pressing sintering. Simlar result had been reported by Huang et al. [3].

### 3.2. Stock Utilization

**Figure 4:**
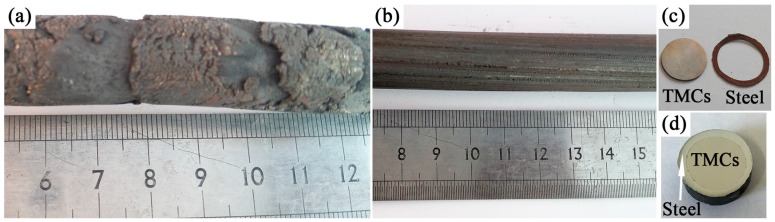
As-extruded composites extruded without using steel can (**a**) and using steel can (**b**–**d**).

Figure 4 showed the images of the as-extruded TiBw/TC4 composites extruded without using steel can and using steel can. It was clearly seen in Figure 4a that the surface of the composites extruded without steel can had deep cracks. That was because although the temperature of the composites was 1150 °C at the time of extrusion, the temperature of the mold was only 300 °C. When the composites were extruded, the surface temperature of the composites decreased rapidly resulting in the increase of the deformation resistance of the composites. The surface of the composites adhered to the mold, which could make the disharmonious deformation of the inside and outside of the composites appear. The disharmonious deformation would lead to the deep cracks on the surface of the composites. 

When the 45# steel can was used during the extrusion process, the surface of the composites (Figure 4b) was smoother than that extruded without using can. The interface between the composites and the steel was very clear and no interface reaction occurred (Figure 4c,d). The circular degree of the composites was high, which meant that the composites and steel underwent harmonious deformation during the extrusion process. The high temperature deformation resistance of 45# steel was much smaller than that of the composites and the steel can played the role of lubricant during the extrusion process. The steel could be removed by centerless grinding and the utilization rate of as-extruded composites could be more than 90%. For the composites extruded without can, only the core part of the composites bars could be considered as effective part and the utilization rate dropped to 60–70%. It meant that it was possible to improve the utilization rate of the composites by using the canned extrusion method for fabricating as-extruded composites bars.

### 3.3. Microstructure Observation

**Figure 5:**
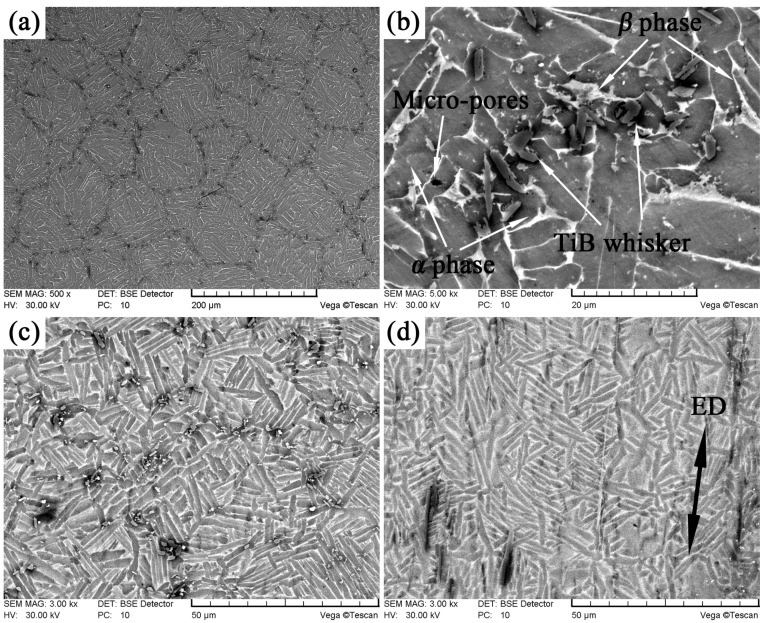
SEM images of the as-sintered composites ((**a**) low magnification; (**b**) high magnification) and as-extruded composites ((**c**) cross section; (**d**) longitudinal section).

Figure 5 shows the microstructure of the as-sintered and as-extruded 2.5 vol % TiBw/TC4 composites. It was clearly seen in Figure 5a,b that the TiB whiskers were successfully synthesized by the reaction of TiB_2_ and Ti surrounding TiB_2_ during the vacuum hot-pressing sintering process. The in-situ synthesized TiB whiskers were distributed around the original spherical TC4 powders to form a 3D equiaxed network and similar results had been reported by Huang et al. in the TiBw/Ti60 composites [12]. It could also be said that TiB whiskers were distributed at the grain boundary of primary β grain, forming a quasi-continuous network and the orientation of the TiB whisker was random. The size of the network was consistent with the size of the original spherical titanium powder. It implied that the sintering process was a process of bridging the interface and no obvious deformation had occurred. The reason for the network distribution of TiB whisker was that during the low energy milling process, the small TiB_2_ powders adhered to the large-sized spherical titanium powders. This result could be confirmed by the size of the TC4 powders and mixed powders measured by laser particle size distribution instrument. The sizes of the original TC4 powders and mixed powders were 115 µm and 125 µm, respectively. It revealed that the low energy milling did not break up the large spherical TC4 powders but enabled the small-sized TiB_2_ to adhere onto the surface of the TC4 powders. During the sintering process, TiB was synthesized through the in-situ reaction between TiB_2_ and nearby titanium and TiB was whisker-like (Figure 5b) due to its special B27 structure [12]. The matrix of the composites emerged a typical α + β structure presenting laminal α phase and intergranular β phase. The α phase was dark, while the residual β phase was the lighter constituent. The α clusters were very coarse due to the low furnace cooling rate. It should also be noted that there were a small number of micro-pores in the matrix and the size of them was less than 2 µm. The presence of these micro-pores would not become the starting source of the crack and therefore did not adversely affect the properties of the composites [20].

After extrusion, not only the microstructure of the matrix but also the distribution of the TiB whisker had undergone significant changes. Since the TiB_2_ had been completely consumed during the hot-pressing sintering process, only the distribution and orientation of whiskers were changed during the extrusion process and there was no formation of whiskers. In the cross section (Figure 5c), the TiB whisker was also distributed in the form of 3D equiaxed network as that in the as-sintered composites (Figure 5a). It was worth noting that the size of euqiaxed network was changed to approximately 40 µm in the as-extruded composites from 120 µm in the as-sintered composites. That was because compression deformation occurred in the cross section to shrink the network and the shrinkage degree was consistent with the size of original network in as-sintered composites divided by the transverse section deformation amount. There was no obvious difference in matrix microstructure except for the size of the grain. The grain was obviously refined in the as-extruded composites. The reason was that the primary β grain also shrank in the cross section. In the subsequent phase change (β → α) process, the size of α precipitate and residual β phase decreased due to the reduction of the size of primary β grain. Moreover, the faster cooling speed after extrusion (air cooling) limited the growth of grain [21].

In the longitudinal section of the as-extruded composites (Figure 5d), the orientation of TiBw was aligned along the extrusion direction. During the extrusion process, the TiB whisker twisted and after extrusion their longitudinal axes were parallel to the extrusion direction. The microstructure evolution was discussed in the following section. The pre-sintering temperature was 1150 °C (above the β transus) and the sintering time was 0.5 h, and at this time the matrix had all become β phase. The size of β grain was consistent with the size of network (i.e., the size of the original spherical titanium powders) due to the limit of the TiB whisker. The primary β grain was elongated in the longitudinal section during the extrusion process. The completely dynamic recrystallization (DRX) occurred because of the high energy and large deformation to form the equiaxed primary β grain in the ellipsoidal network. Because the time of extrusion and cooling process was very short, these grains had no time to grow up. Part of whiskers would break during the extrusion process. However, there were on obvious cracks in the composites. That was because the deformation time was very short and the deformation degree was large, the cracks produced by the fractured TiB whiskers were remedied by the rapid deformation matrix due to the 3D compressive stress in the extrusion process [19]. There was another phenomenon which was needed to be pointed out that the connectivity of the matrix became better after extrusion. TiB whiskers were distributed in the form of network before extrusion like cells on the matrix. Although the grain boundary could be strengthened at this time, the connectivity of the matrix deteriorated. After extrusion, the composites underwent elongation deformation in the longitudinal section and at this time the distribution of whiskers would become more dispersed [19], resulting in the better connectivity of the matrix. It was indirectly implied that the ductility of the as-extruded composites was better than that of the as-sintered composites.

**Figure 6:**
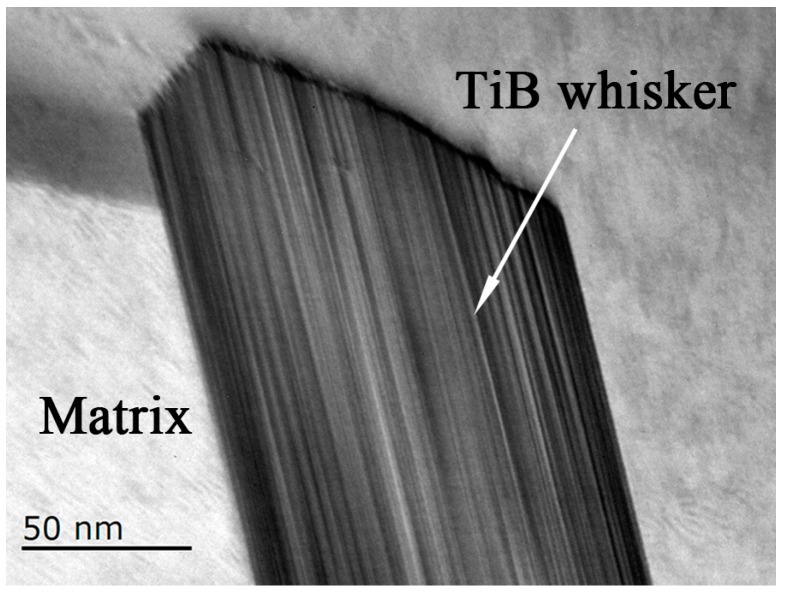
The bright-filed TEM image of the interface between the matrix and TiB whisker in as-extruded composites.

Figure 6 shows the bright-field image of the interface of the TiB whisker and the matrix in as-extruded composites. It could be clearly seen that the interface between the matrix and the in-situ synthesized TiB whisker was clean and no new precipitation, reagent and micro-pore could be observed. This result indicated that although the whiskers twisted during the extrusion process, the interface was still well combined. Moreover, no new reaction occurred during the extrusion process. 

Almost all the literatures studying on the extrusion of the TiB whisker-reinforced TMCs [18,19,22,23] expressed that the whiskers would break during the subsequent hot deformation process. However, there was no discussion about which parts were liable to rupture. In our previous work on the TiB whisker-reinforced TA15 matrix composites [20], the TiB whiskers, whose longitudinal axes were parallel to the tensile load direction, were prone to break during the tensile testing process. In the extrusion process, the TiB whiskers whose longitudinal axes were inconsistent with the extrusion direction would twist with the flow of the matrix and this part of the whiskers was mainly subjected to shear. So, it was less likely that this part of the whiskers would break. It could also be confirmed by the phenomenon that only the debonding between TiB whisker and matrix occurred but not the fracture of TiB whisker for the TiB whisker whose longitudinal axes were perpendicular to tensile load direction during the tensile testing process of the TiB whisker-reinforced TA15 composites fabricated by vacuum hot-pressing sintering [20]. The whiskers whose longitudinal axes were parallel to the extrusion direction were subjected to tensile stress during the extrusion process and were more prone to break. It was similar to the fracture of the TiB whisker during the tensile testing process of the TiB whisker-reinforced TA15 matrix composites fabricated by powder compact extrusion [24].

### 3.4. Mechanical Properties

**Figure 7:**
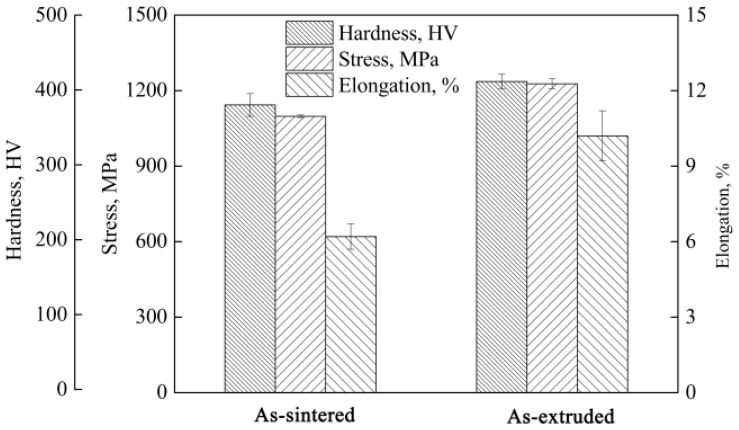
Room temperature mechanical properties of the as-sintered and as-extruded composites.

Figure 7 shows the mechanical properties of the as-sintered and as-extruded composites. All properties of the composites, ultimate tensile strength, Vickers hardness and ductility, increased after extrusion. The ultimate tensile strength and fracture elongation of the composites were increased from 1098 MPa, 6.2% to 1227 MPa, 10.2%, respectively, along the extrusion direction. That was to say, the improvement of the ultimate tensile strength and fracture elongation due to extrusion reached to 11.7%, 65%, respectively. Compared to the 5 vol % TiC particle reinforced TC4 matrix composites, fabricated by casting (1005 MPa, 3.61%) [25], the as-extruded 2.5 vol % TiB whisker-reinforced TC4 matrix composites showed an obvious improvement in tensile properties. Hardness increased due to the grain refinement caused by the extrusion. The improvement in ultimate tensile strength was mainly attributed to two aspects. Firstly, after extrusion complete DRX occurred in the matrix and the grains did not have enough time to grow up, resulting in the grain refinement in the matrix. The reinforcement effect of this part could be explained by the well-known Hall-Petch relationship [26,27]. Secondly, the directional distribution of whiskers would increase the strength in this direction. It could be expressed as the following equation [28,29].

(1) $$∆\sigma_{\mathrm{TiB}}=0.5\sigma_{0.2}\;V_{\mathrm{TiB}}l/d\;\omega$$

Here ∆σ_TiB_ was the yield strength of the composites, σ_0.2_ the yield strength of the matrix, V_TiB_ the volume fraction of TiB whisker, *l*/*d* the aspect ratio of TiB whisker, and ω the whisker orientation factor. As can be seen in Figure 5d, in the as-extruded TMCs, TiB was oriented with their longitudinal axes parallel to the extrusion direction, so ω was close to 1 (the largest value) in this situation. Additionally, it could be seen in Figure 5d that some of the TiB whiskers were broken into several sections during the extrusion process leading to the decrease in aspect ratio (*l*/*d*). According to 19, the effect of aspect ratio decrease on the tensile strength was much less than the effect of the orientation of TiB whisker (1/0.27 to 0.08). In the as-sintered composites, the TiB whisker was in a random array model and ω = 0.27 [28,29]. Therefore, the effect of aspect ratio on strength could be ignored. The reason for the improvement in ductility was that in as-sintered composites, the network structure of the whiskers was like a shell covering the matrix. This structure would limit the connectivity of the matrix leading to a poor ductility. After extrusion, the shell structure was destructive and the connectivity of the matrix became better. These results indicated that the mechanical properties of the as-sintered composites could be further improved by hot extrusion deformation.

### 3.5. Fractography

**Figure 8:**
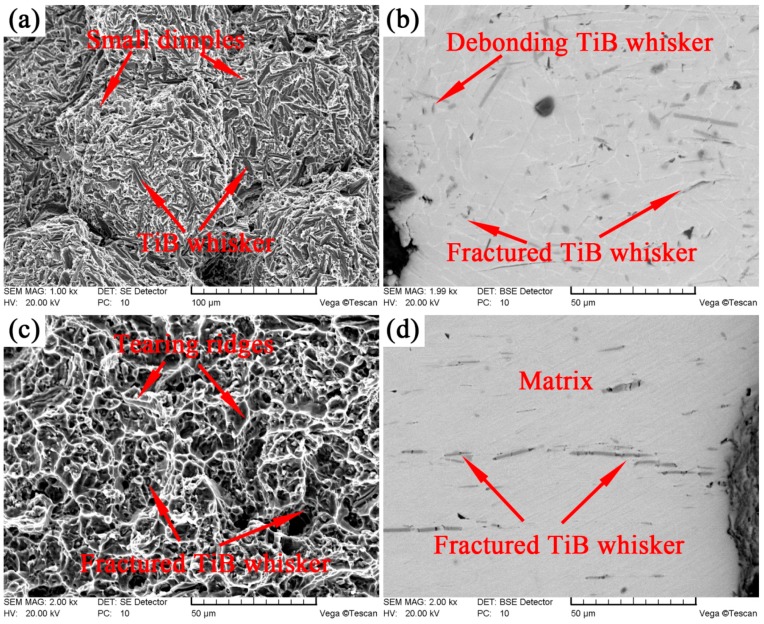
SEM images of the tensile test specimen: (**a**,**b**) as-sintered composites; (**c**,**d**) as-extruded composites; (**a**,**c**) fracture surface; (**b**,**d**) longitudinal sections near the fracture surface.

In order to better understand the deformation behavior and the strengthen effect of TiB whisker in as-sintered and as-extruded composites, as well as the fracture mechanisms at room temperature, fracture surfaces and longitudinal sections near the fracture surfaces of the fractured samples were examined (as shown in Figure 8). The fracture was concentrated at the network boundary for the as-sintered composites (Figure 8a,b). There were TiB whisker and matrix on the fracture surface due to the quasi-continuous characteristic of the network. The debonding between the TiB whisker and the matrix and fracture of TiB whisker formed the cleavage facets in Figure 8a. There were many shallow dimples in the matrix through the fracture surface indicating a certain ductility of the composites and this was consistent with the mechanical properties (Figure 7). These results indicated that the fracture mechanism of the as-sintered composites was a mixture of brittle cleavage fracture (TiB whisker) and ductile failure (matrix).

It could be seen in Figure 8c,d that almost all whiskers near the fracture surface fractured after tensile test indicating the better strengthen effect of the TiB whisker in as-extruded composites. Moreover, this result also illustrated the excellent debonding force between the matrix and the TiB whisker although most of them twisted with the flow of the matrix during the extrusion process, which was in agreement with the bright-filed TEM image (Figure 6). It was also consistent with the result of the higher ultimate tensile strength of the as-extruded composites. There were many dimples and tearing ridges through the fracture surface. These results indicated that the fracture mechanism of the as-extruded composites was also a mixed fracture. It should be noted that the dimples and tearing ridges were much larger and deeper than that in the as-sintered composites (Figure 8a), which indicated the as-extruded composites underwent larger deformation before fracture failure. This result was consistent with the result of the better ductility of the as-extruded composites (Figure 7).

It could be seen in Figure 8b that only parts of the TiB whiskers fractured. Specifically, the whiskers parallel to the load direction were broken and the whiskers perpendicular to the load direction were not broken. A conclusion might be drawn that during the deformation process of the composites, the strengthen effect of the TiB whiskers mainly came from whiskers parallel to the tension load. In other word, the smaller the angle between the axis of TiB whisker and the load direction were, the better the strengthen effect was. These results were consistent with the Equation (1).

## 4. Conclusions

In this work, the effects of extrusion on the stock utilization, microstructure and mechanical properties of composites which were extruded whether using the steel can or not were investigated. Main conclusions were drawn as follows:

(1) When the composites were extruded without steel can, the surface of the composites had deep cracks due to the disharmonious deformation of the inside and outside of the composites. When the composites were extruded with steel can, the stock utilization could reach more than 90% and increase by 20~30% compared with the composites extruded without steel can.
(2) After extrusion, the mechanical properties of the composites had an obvious improvement. The improvement was caused by grain refinement, orientation distribution of the whiskers and better connectivity of the matrix.
(3) The TiB whiskers were randomly distributed in the as-sintered composites but oriented in the as-extruded composites. During the extrusion process, the TiB whiskers whose longitudinal axes were inconsistent with the extrusion direction would twist to the extrusion direction with the flow of the matrix and the whiskers whose longitudinal axes were consistent with the extrusion direction were prone to break.
